# Implementing a robotic liver resection program does not always require prior laparoscopic experience

**DOI:** 10.1007/s00464-021-08645-1

**Published:** 2021-10-04

**Authors:** Emanuele Balzano, Lorenzo Bernardi, Giovanni Tincani, Davide Ghinolfi, Fabio Melandro, Jessica Bronzoni, Sonia Meli, Giuseppe Arenga, Giandomenico Biancofiore, Laura Crocetti, Paolo De Simone

**Affiliations:** 1 Hepatobiliary Surgery and Liver Transplantation, University of Pisa Medical School Hospital, Pisa, Italy; 2 Intensive Care Unit, University of Pisa Medical School Hospital, Pisa, Italy; 3 Interventional Radiology, University of Pisa Medical School Hospital, Pisa, Italy; 4 Department of Surgical, Medical, Biochemical Pathology and Intensive Care, University of Pisa, Pisa, Italy

**Keywords:** Hepatocellular carcinoma, Liver resection, Robot, Learning curve, Outcomes

## Abstract

**Background:**

Preliminary experience in laparoscopic liver surgery is usually suggested prior to implementation of a robotic liver resection program.

**Methods:**

This was a retrospective cohort analysis of patients undergoing robotic (RLR) versus laparoscopic liver resection (LLR) for hepatocellular carcinoma at a center with concomitant initiation of robotic and laparoscopic programs

**Results:**

A total of 92 consecutive patients operated on between May 2014 and February 2019 were included: 40 RLR versus 52 LLR. Median age (69 vs. 67; *p* = 0.74), male sex (62.5% vs. 59.6%; *p* = 0.96), incidence of chronic liver disease (97.5% vs.98.1%; *p* = 0.85), median model for end-stage liver disease (MELD) score (8 vs. 9; *p* = 0.92), and median largest nodule size (22 vs. 24 mm) were similar between RLR and LLR. In the LLR group, there was a numerically higher incidence of nodules located in segment 4 (20.0% vs. 16.6%; *p* = 0.79); a numerically higher use of Pringle’s maneuver (32.7% vs. 20%; *p* = 0.23), and a shorter duration of surgery (median of 165.5 vs. 217.5 min; *p* = 0.04). Incidence of complications (25% vs.32.7%; *p* = 0.49), blood transfusions (2.5% vs.9.6%; *p* = 0.21), and median length of stay (6 vs. 5; *p* = 0.54) were similar between RLR and LLR. The overall (OS) and recurrence-free (RFS) survival rates at 1 and 5 years were 100 and 79 and 95 and 26% for RLR versus 96.2 and 76.9 and 84.6 and 26.9% for LLR (log-rank *p* = 0.65 for OS and 0.72 for RFS).

**Conclusions:**

Based on our results, concurrent implementation of a robotic and laparoscopic liver resection program appears feasible and safe, and is associated with similar oncologic long-term outcomes.

Liver resection is the backbone of the management of hepatocellular carcinoma (HCC), with the potential for radicality in early-stage tumors and well-selected candidates [1]. While open liver resections (OLR) have largely expanded in the Nineties, minimally invasive liver surgery (MILS)—such as robotic (RLR) and laparoscopic liver resections (LLR)—have been refined only more recently [2, 3]. The advantages of MILS have largely been described in the literature, and MILS has gained wider consent among health care professionals [4, 5]. Compared to open surgery, RLR and LLR are associated with less intraoperative blood losses, lower rates of complications, and shorter length of postoperative stay due to faster patient recovery [6, 7]. In particular, MILS is associated with a reduced risk of postoperative ascites and liver failure in patients with cirrhosis [8], while providing comparable oncological outcomes to OLR [5, 7, 9].

Superiority of either MILS technique has not yet been confirmed [10, 11]. Similar safety and oncologic outcomes have been reported [12, 13], but the robotic approach allows to overcome some technical limitations of laparoscopy, such as access to posterior and superior liver segments, which may be technically demanding in LLR [14–16]. Finally, the learning curve seems to be shorter for RLR when compared to laparoscopy [17, 18].

Usually, robot-assisted surgery programs are implemented only after experience in laparoscopy has been achieved [19]. This was not the case at our department, where a robotic liver resection program was implemented concomitantly with laparoscopic liver surgery. The aim of the current paper is to illustrate the results of our initial experience with RLR versus LLR, discuss the feasibility of a robotic liver surgery program without prior experience in major laparoscopic liver surgery, and present the safety and oncological efficacy of both the procedures for the treatment of HCC.

## Materials and methods

### Study design and setting

This was a retrospective cohort analysis of consecutive patients undergoing RLR versus LLR for the treatment of HCC at the Hepatobiliary surgery and Liver transplantation Unit of the University of Pisa Medical School Hospital between May 2014 and February 2019. Data were derived from a prospectively collected database and retrospectively reviewed with focus on the characteristics of the population, tumors, and surgical procedures. The primary endpoint of our analysis was to assess the feasibility, safety, and oncological efficacy of both RLR and LLR as per conversion rates, incidence of complications, and overall (OS) and recurrence-free survival (RFS) rates. Type and severity of complications were classified according to Dindo–Clavien [20]. Blood transfusions included the intraoperative and postoperative period. We also assessed the distribution of complications per surgeon and population quartiles to account for the individual and institutional learning curve.

### Indications to surgery

The preoperative diagnosis of HCC was according to the European Association for the Study of the Liver (EASL) guidelines [21, 22]. In the current experience, RLR and LLR were both reserved to patients with compensated chronic liver disease (i.e., Child–Pugh A or B cirrhosis), tumors within Milan criteria and/or lesions not amenable to local ablative treatment (as per subcapsular site or proximity with major vascular and biliary structures). In contrast with the most recent recommendations, the presence of portal hypertension (i.e., either splenomegaly associated with platelet count < 100,000/mm^3^ or esophageal varices) was not considered an exclusion criterion to either procedure [21]. Chronic treatment with antiplatelet drugs was not interrupted before surgery.

### Implementation of the surgical program

Prior to initiation of the current programs, our senior surgical staff (EB, GT, DG, FB, GC, GA, PDS) had achieved experience in minor laparoscopic procedures (cholecystectomy; appendectomy), non-resective liver surgeries (i.e., cyst fenestration), and colic resection. Implementation of RLR and LLR was preceded by participation of all surgeons to accredited international programs, and the first 10 robotic procedures were tutored by a senior surgeon from an external institution. No external mentoring was performed for LLR.

### Statistical analysis

According to their level of measurement and distribution, continuous variables were expressed as means and standard deviations (SD) or medians and ranges, while categorical variables are described as frequencies. Data were compared with the t-test for continuous values with normal distribution, the Mann–Whitney *U* test for continuous values without normal distribution, and the Pearson’s chi-square or Fisher’s exact tests for categorical values. The level of significance was set at 5%. Survival rates were obtained according to Kaplan–Meier at 1, 2, and 5 years after surgery, and comparison between group was according to the log-rank. Survival was censored at death, latest follow-up, or at transplantation in the event of salvage liver transplantation (LT). The latter was indicated in the case of HCC recurrence or liver function decompensation. The study protocol was approved by the Institutional Review Board of the University Hospital of Pisa, Italy. The patients provided written informed consent to current analysis.

## Results

A total of 92 patients were included: 40 underwent RLR as opposed to 52 treated with LLR. The demographic and clinical characteristics of interest of the 2 groups are illustrated in Table 1. No statistically significant difference was observed for any of the clinical variables (Table 1). There was a numerically higher incidence of previous abdominal surgeries and antiplatelet use for RLR versus LLR, and a numerically higher frequency of nodules located in segment 4 for LLR patients (Table 1).

**Table 1:** Demographic and clinical characteristics of the study populations

Variable	Robotic (#40)	Laparoscopic (#52)	*p*
Male, *n* (%)	25 (62.5)	31 (59.6)	0.96
Age (years), median [range]	69 [46–83]	67 [34–86]	0.74
BMI (Kg/m^2^), median [range]	26.0 [15.2–34.8]	26.4 [18.0–33.6]	0.98
ASA score, median [range]	3 [1–4]	3 [1–4]	0.99
Previous abdominal surgery, *n* (%)	18 (45.0)	15 (28.8)	0.10
Antiplatelet treatment, *n* (%)	9 (22.5)	6 (11.5)	0.15
Cirrhosis	39 (97.5)	51 (98.1)	0.85
MELD score, median [range]^a^	8 [6–13]	9 [6–15]	0.92
Total nodules, *n*	43	55	
Nodules per patient, median [range]	1 [2]	1 [3]	0.98
Largest nodule diameter (mm), median [range]	22 [12–65]	24 [11–58]	0.97
Tumor nodule site, *n* (%)			
S2	2 (4.7)	4 (7.4)	0.69
S3	6 (14.0)	8 (14.5)	0.99
S4	7 (16.3)	11 (20.0)	0.79
S5	8 (18.6)	10 (18.2)	0.99
S6	9 (20.9)	14 (25.4)	0.63
S7	2 (4.7)	0 (0)	0.19
S8	9 (20.9)	8 (14.5)	0.43

*ASA* American Society of Anesthesiology, *MELD* model for end-stage liver disease

^a^MELD is provided for patients with cirrhosis

The total number of treated lesions was 98 (43 for RLR vs. 55 for LLR), and the median largest nodule diameter was 22 and 24 mm for RLR vs. LLR (Table 1). Surgical procedures are reported in Table 2 and consisted mainly of wedge resections (90% for RLR vs. 84.6% for LLR, *p* = 0.44). To note, use of Pringle’s maneuver was numerically more frequent in LLR (32.7% vs. 20.0; *p* = 0.23), and median duration of surgery was significantly longer for RLR versus LLR (217.5 vs. 165.5 min; *p* = 0.04) (Table 2). Cholecystectomy was associated in 4 (10.0%) RLR patients versus 9 (16.4%) LLR, while one (2.5%) RLR patient underwent simultaneous resection of a suspected tumor nodule located in the right kidney upper pole.

**Table 2:** Surgical data

Procedure	Robotic (#40)	Laparoscopic (#52)	*p*
Wedge resection, *n* (%)	36 (90.0)	44 (84.6)	0.44
Segmentectomy, *n* (%)	2 (5.0)	4 (7.7)	0.69
Bisegmentectomy, *n* (%)	2 (5.0)	4 (7.7)	0.69
Pringle’s maneuver, *n* (%)	8 (20.0)	17 (32.7)	0.23
Length (min), median [range]	217.5 [95.0–390.0]	165.5 [80.0–256.0]	0.04
Conversion, *n* (%)	3 (7.5)	2 (3.8)	0.64
Switch to ablation, *n* (%)	2 (5.0)	0 (0)	0.18
Patients transfused, *n* (%)	1 (2.5)	5 (9.6)	0.21
Blood loss < 200 mL, *n* (%)	39 (97.5)	47 (90.4)	0.22
R0, *n* (%)	36 (90.0)	46 (88.5)	0.99
LOS (days), median [range]	6 [3–15]	5 [2–13]	0.54
Patients with complications^a^, *n* (%)	10 (25.0)	17 (32.7)	0.49
Total complications^a^, *n*	13	21	
Grade 1	9	13	0.72
Grade 2	1	3	0.99
Grade 3	2	3	0.99
Grade 4	1	2	0.99

*LOS* length of stay

^a^As per Dindo–Clavien

The conversion rate was numerically higher for RLR (7.5% vs. 3.8%; *p* = 0.64), while in 2 (5.0%) RLR patients with one nodule each the surgeon decided to switch the strategy from resection to radiofrequency ablation due to the presence of ascites. In the RLR group, reasons for conversion to open surgery were bleeding in 2 (5.0%) cases (one from the liver parenchyma, and one from the combined kidney resection margin), and oxygen desaturation in one (2.5%). Both conversions (3.8%) in the LLR group were due to bleeding from the liver cut surface. Blood transfusions were numerically higher for LLR versus RLR (9.6% vs. 2.5%; *p* = 0.21).

In the RLR group, histology of 41 resected specimens was consistent with HCC in 39 (90.7%), cholangiocarcinoma in one (2.3%), and a fully necrotic nodule in a further one (2.3%). In the LLR group, histology showed HCC in 54 (98.2%) nodules, and mixed HCC-cholangiocarcinoma in one (1.8%). R0 was achieved in 90.0% and 88.5% of RLR and LLR patients, respectively (*p* = 0.99) (Table 2). The median postoperative length of stay was 6 days for RLR as opposed to 5 for LLR (*p* = 0.54) (Table 2).

During the hospital stay, 10 (25.0%) RLR patients presented a total of 13 complications versus 17 (32.7%) LLR patients with 21 complications (*p* = 0.49). Grade ≥ III complications were observed in 7.5% RLR patients and consisted of one hematoma of the muscle rectus abdominis due to parietal bleeding at the level of the trocar port site and requiring radiology-guided embolization, ascites requiring percutaneous drainage, and surgical site active bleeding requiring open redo surgery (Table 2). Five (9.6%) LLR patients presented grade ≥ III complications: two cases of ascites necessitating percutaneous drainage, two cases of abdominal collection requiring percutaneous drainage, and one surgical site active bleeding requiring open redo surgery (Table 2).

Median number of patients with complications per staff surgeon was 2 (range, 1–3) in the RLR group versus 2.5 (range 1–3) in the LLR group (*p* = 0.78). Patient quartile breakdown analysis did not show any difference in the distribution of complications in any of the 2 groups. Namely, in the RLR group, the frequency of patients with complications was 3 (7.5%), 2 (5.0%), 3 (7.5%), and 2 (5.0%) in the first, second, third, and fourth quartiles (*p* = 0.98) versus 5 (9.6%), 4 (7.7%), 4 (7.7%), and 4 (7.7%) in the LLR group (*p* = 0.99).

In the RLR group, the median follow-up was 24 months (range, 8–57): 16 (40.0%) patients presented HCC recurrence, one (2.5%) such patient died due to recurrence, 2 (5.0%) patients died for progression of liver disease, and one patient was lost to follow-up. Four (10.0%) patients underwent salvage LT due to HCC recurrence and the median time from resection to LT was 8 months (range, 5–10).

In the LLR group, the median follow-up was 27.5 months (range, 9–55): 18 (34.6%) patients presented HCC recurrence, 4 (7.7%) such patients died to recurrence, 4 (7.7%) died of progression of liver disease, and 2 (3.8%) died of sepsis 15 and 18 months after surgery, respectively. Five (9.6%) patients underwent salvage LT due to HCC recurrence and the median time from resection to LT was 11 months (range, 4–13).

**Fig. 1:**
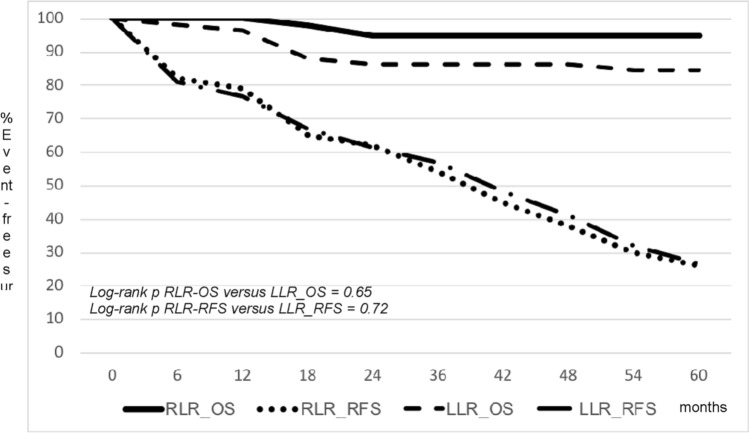
Kaplan–Meier estimates of overall (OS) and recurrence-free survival (RFS) in robotic (RLR) versus laparoscopic (LLR) liver resection for hepatocellular carcinoma

The actuarial 1-, 2-, and 5-year OS and RFS were 100 and 79%; 95 and 62%; and 95 and 26.0% for RLR patients versus 96.2 and 76.9%; 86.5 and 61.5%; 84.6 and 26.9% for LLR (log-rank *p* = 0.65 for OS and 0.72 for RFS). (Fig. 1).

## Discussion

With this retrospective cohort analysis, we aimed to evaluate the results of our initial experience with MILS for HCC, whether it be in the form of RLR or LLR. Unlike the majority of robot-assisted surgery programs, which are usually implemented once solid experience with LLR has been achieved, our MILS program consisted of concomitant implementation of either technique. Based on current results, preliminary experience with LLR might not be a necessary pre-requisite to robot-assisted liver surgery, although some requirements are to be complied with, such as extensive experience in open liver surgery and preliminary training in both the MILS techniques.

From a technical standpoint, however, some differences between RLR and LLR have to be underscored. The 7-degree motion of robotic arms provides excellent dexterity, and allows easier access to posterior and superior liver segments (S7, S8, S4b) when compared to LLR [16]. This might account for the fact that, despite similar prevalence of cirrhosis and concomitant medication with antiplatelet drugs in both groups, hilar clamping was necessary only in 20% of cases of RLR patients versus 32.7% for LLR. In the remainder of RLR patients, it was possible to perform clamp-free parenchymal dissection with very low transfusion rate (2.5%) and non-significant blood losses (< 200 mL) in all cases but one. The increased level of dexterity of robot-assisted surgery might also explain why previous open abdominal surgery—which was reported in 45% (18/40) of RLR patients—did not seem to affect its feasibility or safety rates of RLR, nor its oncological efficacy. In such cases, careful dissection of adherences could be carried out and inadvertent visceral injuries were avoided. On the opposite, it is important to note that RLR was a longer procedure than LLR, since duration of surgery is influenced by preliminary (docking) phases and device repositioning during the varied phases of surgical procedures. No major impact was observed on length of hospital stay, which was a median of 6 and 5 days, respectively, for RLR and LLR. This might reflect the initial learning curve phase of our institution, since no difference was observed between the two cohorts.

Even if the present study elucidated the absence of differences in terms of feasibility or safety of RLR versus LLR for the treatment of HCC, it did not allow to clarify the duration of each individual surgeon learning curve with either technique. The learning curve is a rather complex process which is influenced by surgeon, patient, and institution-derived correlates and it deserves properly powered studies and investigations. Our experience was biased by the concomitant practice of open liver surgery and transplantation, as well as by the level of efficiency achieved by the anesthesiology and nursing teams. All of these variables, alongside a detailed case-mix analysis, should be included in future trials to unveil the intricacies underlying the learning curve effect.

Despite the technical differences between RLR and LLR, their oncologic efficacy is comparable. In both populations, we achieved similar overall and recurrence-free survival rates, and the majority of recurrences occurred 2 years after surgery in either group. These data are consistent with the high prevalence of cirrhosis among our patients. However, OS plateaued 2 years after surgery, thanks to implementation of treatment strategies for HCC recurrences (data not shown), including radiologic treatments, oral drugs, and ultimately LT [1]. Of interest, 4 (10.0%) RLR and 5 (9.6%) LLR patients underwent salvage LT at a median interval of 8 and 11 months after primary surgery, respectively. To this regard, Guerrini et al. have reported that salvage LT is a viable treatment option for local recurrences, and that previous RLR does not seem to impair the long-term outcome of transplantable cirrhotic patients affected with recurrent HCC [23]. However, only a third of these patients can benefit from salvage transplantation, suggesting that patient selection for a “resection-first” policy together with prompt recurrence detection are both key to improving the results of liver resection followed by salvage LT [24]. Based on the low rate of complications and the comparable oncological efficacy of MILS versus open surgery, RLR and LLR deserve further investigation as bridging modalities prior to LT in order to protect patients from the risk of wait list drop out or as a resection-first strategy followed by salvage LT in the event of local recurrence [25, 26].

In conclusion, our experience confirms that RLR and LLR can be implemented concurrently for the treatment of HCC in compensated cirrhotic patients and in centers with extensive liver surgery practice. Both techniques provide similar efficacy, with the potential to overcome the burden of open surgery and preserve eventual eligibility to transplantation. Based on their advantages, MILS deserves to be investigated as the elective pre-liver transplant bridging modality for those patients with resectable HCC and anticipated longer wait listing times.
